# Challenges and Outcomes of Three Years of the Adhesive Intestinal Obstruction Management Protocol

**DOI:** 10.7759/cureus.98269

**Published:** 2025-12-01

**Authors:** Moataz M Ewedah, Dixon Osilli, Praveen Surya Ravichandran, Tarun Sai Krishna Puli, Faris A Abbadi, Tanya Krasteva, Sayed Haschmat Sarwary

**Affiliations:** 1 Surgery, Barking, Havering and Redbridge University Hospitals NHS Trust, Romford, GBR; 2 Paediatrics and Child Health, Barking, Havering and Redbridge University Hospitals NHS Trust, Romford, GBR

**Keywords:** acute small bowel obstruction, adhesiolysis, asbo, gastrografin, nasogastric tube (ngt), sbo, small-bowel obstruction

## Abstract

Introduction: Small bowel obstruction (SBO) is a significant contributor to surgical morbidity, mortality, and healthcare costs, contributing to a significant number of emergency laparotomies. Advancements in diagnostic techniques have improved the accuracy in delineating simple from complicated SBOs. Some adhesive SBOs will ultimately need surgical intervention, mostly when they are complicated cases. Recently, minimally invasive surgeries, enhanced recovery protocols, and multidisciplinary approaches have become more popular in optimising recovery and minimising recurrence risks. On the other hand, most adhesive SBOs can be managed conservatively with bowel rest, nasogastric (NG) decompression, intravenous fluid therapy, and water-soluble oral contrast agents, leading to a significant reduction in operation-related morbidity, hospital stays, and healthcare costs. Current guidelines recommend a trial of conservative management, although not without controversy.

Aim: This study aimed to explain the predictive factors of success or failure of treatments of adhesive SBOs and statistically report the effect of co-morbidities and expected outcomes of SBO management, based on three-year data from a single high-volume centre in London, UK.

Methodology: Our retrospective-descriptive study included patients with clinical signs of SBO, older than 16 years old, and CT proving adhesive SBO, who required hospital admission. We excluded patients with other causes of SBO and those who had no CT evidence of adhesive disease. Data were collected from online hospital records and analysed in Microsoft Excel® (Microsoft Corporation, Redmond, Washington). Hospital protocol was NG decompression for at least six hours in uncomplicated cases, followed by oral or NG Gastrografin, with abdominal X-rays taken at six and 12 hours post-Gastrografin intake to assess contrast in the colon or rectum. Successful cases were then given a trial of oral feeds before discharge. Those with no contrast in the large intestine, persistent abdominal pain, and onset abdominal tenderness or peritonitis were taken to surgery.

Results and limitations: Out of 415 patients, 30.4% required surgery either as initial mode of treatment or after failed conservative management, proving the effectivity of therapeutic Gastrografin (69.6%), followed by 35.2% being 75 or less years old, proving SBO is more prevalent in elderly population with co-morbidities, as 40.5% are ASA Grade 3, prone to post-operative complications. Surprisingly, COPD patients required surgery more often than those who did not have it, as they desaturate due to distension easily. On the other hand, not much statistical difference is seen in the 30-day mortality rate between the operated and conservatively managed groups.

Conclusion: Our study aligns with the literature to prove that our management protocol is safe. Alongside, the post-operative burden of those undergoing surgery is high; it is vital to identify complex patients for surgery to reduce mortality and morbidity. This goes hand in hand with weighing risks and benefits. Elderly patients carry the risk of major complications plus a higher mortality rate. Timely intervention with therapeutic Gastrografin can minimise the hospital stay and the costs for the NHS owing to long stay post-operatively.

## Introduction

Adhesive small bowel obstruction (ASBO) is a significant contributor to morbidity, mortality, and healthcare costs in emergency surgical settings. The financial burden of ASBO is substantial, with the average cost of surgical treatment per admission amounting to £4,677.41, while conservative management averages £1,606.15 [1]. Between 20% and 30% of patients diagnosed with ASBO ultimately require surgical intervention [2].

According to the Bologna Guidelines (2017) [2,3], patients with partial ASBO who show no signs of strangulation can often be managed non-operatively, with the use of water-soluble contrast medium serving both diagnostic and therapeutic purposes. The World Society of Emergency Surgery (WSES) also recommends that non-operative management (NOM) can be extended for up to 72 hours in the absence of strangulation or peritonitis. If there is no improvement after 72 hours of NOM, surgical intervention is advised.

Surgical methods have progressed, increasingly emphasising minimally invasive techniques. Laparoscopic surgery is becoming more popular due to advantages such as less postoperative pain and faster recovery compared to conventional open surgery. Recent literature highlights the importance of personalised surgical decision-making, taking into account the patient’s overall health, the cause of the obstruction, and any complicating factors. Moreover, there is growing acknowledgment of the significance of postoperative care in managing SBO. Enhanced recovery protocols and multidisciplinary strategies are being adopted to optimise patient recovery and reduce the risk of recurrence. In the UK, small bowel obstruction (SBO) is cited as the cause for 51% of all emergency laparotomies [4]. This illustrates the considerable economic impact and resource demands associated with ASBO cases. Imaging studies have shown that CT scans exhibit a high sensitivity of 96% for detecting ASBO and an accuracy of approximately 90% for predicting strangulation and the necessity for urgent surgery [4,5].

Current guidelines, both local and international, advocate for a trial of conservative management; however, there remains controversy regarding the use of Gastrografin, the duration before confirming treatment failure, and the choice between open or minimally invasive surgical approaches [6]. The American Society of Anesthesiologists (ASA) classification helps us identify certain groups of the population who are at risk when planning for the procedure [7]. A study in the Journal of Gastrointestinal Surgery (2021) highlighted that elderly patients with SBO have a substantially higher mortality risk, with rates sometimes reaching up to 25% in cases with severe complications [8].

Recent advancements in diagnostic techniques, such as high-resolution computed tomography (CT) and point-of-care ultrasonography, have improved the accuracy of SBO diagnoses, allowing for better differentiation between simple and complicated cases. These innovations facilitate more effective surgical planning and help reduce unnecessary operations [9]. Moreover, surgical techniques are evolving, with a growing preference for minimally invasive methods. Laparoscopic surgery is increasingly favoured for its benefits, such as reduced postoperative pain and quicker recovery compared to traditional open surgery.

The aim of this study was to identify and statistically evaluate the clinical and demographic factors, including comorbidities, that influence success or failure of treatment of ASBO and to compare the expected outcomes between conservative and surgical management of ASBO patients, based on our single-centre three-year experience in Barking, Havering and Redbridge University Hospitals NHS Trust (BHRUT), which provides acute surgical services for about 800,000 people.

## Materials and methods

Any patient above 16 years of age, with or without a previous abdominal surgery, presenting with clinical signs of acute intestinal obstruction that required hospital admission, and with a CT showing evidence of ASBO, was included. We excluded patients with other causes of SBO, such as gastrointestinal abnormalities (incarcerated hernias, intussusception, small bowel tumours, closed loop obstruction, stricture, volvulus). We excluded all patients who did not have a CT scan to prove an adhesive cause of intestinal obstruction.

Retrospective data of all patients who presented to the acute surgical unit and were admitted under the surgical team in BHRUT between January 2021 and December 2023 were reviewed. Anonymised data were sourced from online records (EPRO, PACS, Cyberlab, Careflow). All patients needed to meet the inclusion criteria above to be included in our database. The sample size so obtained was substantial for a study of this nature, promoting the validity of our analysis.

Our protocol for management of ASBO, once diagnosis is confirmed clinically and radiologically, is to start gastric decompression with an NGT with two to four hourly suctioning. Gastrografin is to be initiated during the next 24 hours after at least six hours of NGT decompression. This time period was variable in our dataset (averaged at 12 hours), factored by pharmacy, nursing, admission, and feasibility. Follow-up abdominal X-ray is to be done at six and 12 hours after ingestion of Gastrografin to assess if the contrast is reaching the colon. Emergency surgery is then advised in patients who have persistent abdominal pain, or if there is an onset of abdominal tenderness or peritonitis, or if the patient becomes haemodynamically unstable, or if the abdominal plain radiograph does not show contrast product in the colon or the rectum. In other cases, the NGT can be removed, and progressive refeeding can be introduced, starting with a liquid diet, and conservative management is to be continued.

In our study, we noted that a group of patients were not offered Gastrografin either because they were indicated straightforwardly for surgery or due to individual controversies among some consultants. Early surgical intervention was indicated for patients with peritonitis, haemodynamic instability, CT-proven bowel ischaemia, or closed-loop obstruction.

Data were spread into a Microsoft Excel® sheet (Microsoft Corporation, Redmond, Washington). Categorical data were represented as numbers and percentages. Quantitative data were expressed as range (minimum and maximum), mean, standard deviation (SD), and median. The chi-square test was used to compare different groups for categorical variables. Fisher’s Exact Correction for chi-square was used when more than 20% of the cells had expected count less than five. The Student t-test was used for normally distributed quantitative variables to compare between two studied groups. The Mann-Whitney test was used for abnormally distributed quantitative variables to compare two studied groups. Odds ratio (OR) was used to calculate the ratio of the odds and 95% confidence interval of an event occurring in one risk group to the odds of it occurring in the non-risk group. The significance of the results obtained was judged at the 5% level.

## Results

The demographic data of the study population, shown in Table 1, reveal a diverse cohort of patients, with a mean age of 66.28 years and a notable representation of both younger and older individuals. Specifically, 269 (64.8%) of the participants are under the age of 75, while 146 (35.2%) are 75 or older, indicating a significant proportion of older patients who may present with more complex medical issues.

**Table 1:** Demographics of patients SD: standard deviation, Min.: minimum, Max.: maximum, BMI: body mass index, COPD: chronic obstructive pulmonary disease, MI: myocardial infarction, CKD: chronic kidney disease, ASA: American Society of Anaesthesiologists.

Parameter	Total (percentage)
Age (years)	
<75	269 (64.8%)
≥75	146 (35.2%)
Mean ± SD	66.28 ± 17.47
Median (Min–Max)	68.0 (11.0–98.0)
Sex	
Male	173 (41.7%)
Female	242 (58.3%)
Comorbidity	
BMI > 40	14 (3.4%)
Diabetes	62 (14.9%)
COPD	38 (9.2%)
Current smoker	26 (6.3%)
Asthma	34 (8.2%)
MI/stroke	53 (12.8%)
Heart failure	35 (8.4%)
Malignancy	115 (27.7%)
CKD	23 (5.5%)
Immunosuppression	28 (6.7%)
Dementia	9 (2.2%)
ASA	
I	46 (11.1%)
II	146 (35.2%)
III	168 (40.5%)
IV	52 (12.5%)
V	3 (0.7%)
Frailty score	
Mean ± SD	4.47 ± 1.68
Median (Min–Max)	4.0 (1.0–9.0)

Gender distribution shows a higher prevalence of female patients, 242 (58.3%), compared to male patients, 173 (41.7%), which may reflect broader trends in healthcare utilisation among different sexes. The presence of comorbidities further highlights the complexity of this patient population. Notably, 115 (27.7%) have a history of malignancy, and 62 (14.9%) are diabetic. Other significant comorbidities include chronic obstructive pulmonary disease (COPD) in 38 (9.2%) of patients and cardiovascular issues such as myocardial infarction or stroke in 53 (12.8%) and heart failure in 35 (8.4%). This suggests that many patients may have underlying health conditions that could complicate their treatment and management of ASBO.

Table 2 illustrates the distribution of cases in the study, highlighting critical aspects of surgical intervention and associated outcomes for patients with ASBO. Among the 415 patients evaluated, 126 (30.4%) patients required surgical intervention, while the majority, 289 (69.6%) patients, were managed non-operatively. This significant proportion of patients treated without surgery emphasises the potential for conservative management strategies in this clinical context. 

**Table 2:** Distribution of the studied cases according to different parameters (n = 415) HAP: hospital-acquired pneumonia, IV: intravenous, MI: myocardial infarction, DVT: deep vein thrombosis, PE: pulmonary embolism, COVID: coronavirus disease, IR: interventional radiology.

Parameter	Total (Percentage)
Therapeutic Gastrografin	
No	159 (38.3%)
Yes	256 (61.7%)
Operation required	126 (30.4%)
Route	
Open	110 (26.5%)
Laparoscopic	14 (3.4%)
Laparoscopic converted to open	2 (0.5%)
Operation performed	
Not operated	289 (69.6%)
Adhesiolysis only	94 (22.7%)
Small bowel resection	32 (7.7%)
Complications	
Collection	11 (2.7%)
Ileus	20 (4.8%)
Wound infection (requiring antibiotics)	8 (1.9%)
Wound infection (requiring drainage)	8 (1.9%)
HAP (requiring oral antibiotics)	1 (0.2%)
HAP (requiring IV antibiotics)	11 (2.7%)
MI	1 (0.2%)
DVT/PE	0 (0.0%)
COVID	3 (0.7%)
Re-operation	2 (0.5%)
Death	6 (1.4%)
Other	13 (3.1%)
Re-attendance following initial assessment	31 (7.5%)
Operation	13/31 (41.9%)
IR drain	3/31 (9.7%)
Gastrografin	20/31 (64.5%)
Mortality	77 (18.6%)
30-day mortality	31 (7.5%)
1-year mortality	29 (7.0%)

Of those who underwent surgery, open procedures were the most common, comprising 110 (87.3%), while laparoscopic surgeries accounted for only 14 (3.4%). The relatively low rate of laparoscopic interventions may reflect the complexity of the cases presented, which often necessitated open surgery for adequate access and treatment. Additionally, the conversion rate from laparoscopic to open surgery for 2 (1.6%) patients indicates that while laparoscopic techniques were attempted, they were not always feasible, underscoring the challenges in managing ASBO. The types of operations performed reveal that adhesiolysis was the most frequent procedure, conducted in 94 (22.7%) cases, followed by small bowel resection in 32 (7.7%).

Table 2 shows a clear distribution of the patient population, with a strong preference for therapeutic Gastrografin among the majority of cases. The data indicate that while a significant number of patients received this treatment, a notable portion required surgical intervention. Understanding the relationship between therapeutic Gastrografin use and the need for surgery could help refine clinical guidelines and optimise patient management strategies in future practice. Further analysis of the outcomes associated with each treatment approach would be beneficial in elucidating the efficacy and appropriateness of Gastrografin in various clinical contexts.

SBO in the elderly group

There are various factors that affect and complicate the presentation, diagnosis, and management of ASBO. Such factors are not the same in the elderly (age ≥75). These are discussed below and are presented in Tables 3, 4.

**Table 3:** Relation between age and different parameters (n = 415) Test of sig.: test of significance, SD: standard deviation, U: Mann–Whitney test, Min.: minimum, Max.: maximum, BMI: body mass index, c2: chi-square test, FE: Fisher exact, COPD: chronic obstructive pulmonary disease, MI: myocardial infarction, CKD: chronic kidney disease, ASA: American Society of Anaesthesiologists.

Parameters	Age <75 Years (Percentage) (n = 269)	Age ≥75 Years (Percentage) (n = 146)	Test of sig.	p-value
Length of hospital stay (days)				
Mean ± SD	8.79 ± 9.73	12.61 ± 14.77	U = 15482.5	0.001*
Median (Min–Max)	6.0 (0.0–80.0)	7.0 (0.0–106.0)		
Sex				
Male	126 (46.8%)	47 (32.2%)	c2 = 8.354*	0.004*
Female	143 (53.2%)	99 (67.8%)		
Comorbidity				
BMI > 40	9 (3.3%)	5 (3.4%)	c2 = 0.002	FEp = 1.000
Diabetes	44 (16.4%)	18 (12.3%)	c2 = 1.208	0.272
COPD	16 (5.9%)	22 (15.1%)	c2 = 9.464*	0.002*
Current smoker	20 (7.4%)	6 (4.1%)	c2 = 1.782	0.182
Asthma	24 (8.9%)	10 (6.8%)	c2 = 0.540	0.462
MI/stroke	26 (9.7%)	27 (18.5%)	c2 = 6.620*	0.010*
Heart failure	13 (4.8%)	22 (15.1%)	c2 = 12.839*	>0.001*
Malignancy	64 (23.8%)	51 (34.9%)	c2 = 5.862*	0.015*
CKD	10 (3.7%)	13 (8.9%)	c2 = 4.863*	0.027*
Immunosuppression	21 (7.8%)	7 (4.8%)	c2 = 1.365	0.243
Dementia	1 (0.4%)	8 (5.5%)	c2 = 11.637*	FEp = 0.001*
ASA				
I	46 (17.1%)	0 (0.0%)	c2 = 58.195*	>0.001*
II	110 (40.9%)	36 (24.7%)		
III	93 (34.6%)	75 (51.4%)		
IV	19 (7.1%)	33 (22.6%)		
V	1 (0.4%)	2 (1.4%)		
Frailty score				
Mean ± SD	3.76 ± 1.39	5.77 ± 1.37	U = 6323.00*	>0.001*
Median (Min–Max)	4.0 (1.0–7.0)	6.0 (2.0–9.0)		

**Table 4:** Relation between age and different parameters (n = 415) SD: standard deviation, U: Mann–Whitney test, c2: chi-square test, FE: Fisher exact, p: probability value for comparison between the studied categories, HAP: hospital-acquired pneumonia, IV: intravenous, MI: myocardial infarction, DVT: deep vein thrombosis, PE: pulmonary embolism, COVID: coronavirus disease, IR: interventional radiology. *Statistically significant at p ≤ 0.05.

Parameter	Age < 75 (Percentage) (n = 269)	Age ≥ 75 (Percentage) (n = 146)	Test of sig.	p-value
Route of operation				
Not operated	189 (70.3%)	100 (68.5%)	c2 = 2.505	FEp = 0.452
Open	67 (24.9%)	43 (29.5%)		
Laparoscopic	11 (4.1%)	3 (2.1%)		
Laparoscopic converted to open	2 (0.7%)	0 (0%)		
Operation performed				
Adhesiolysis	62 (23%)	32 (21.9%)	c2 = 1.126	0.569
Not operated	189 (70.3%)	100 (68.5%)		
Small bowel resection	18 (6.7%)	14 (9.6%)		
Complications				
Collection	9 (3.3%)	2 (1.4%)	c2 = 1.432	FEp = 0.342
Ileus	11 (4.1%)	9 (6.2%)	c2 = 0.888	0.346
Wound infection (requiring antibiotics)	5 (1.9%)	3 (2.1%)	c2 = 0.019	FEp = 1.000
Wound infection (requiring drainage)	6 (2.2%)	2 (1.4%)	c2 = 0.371	FEp = 0.718
HAP (requiring oral antibiotics)	1 (0.4%)	0 (0.0%)	c2 = 0.544	FEp = 1.000
HAP (requiring IV antibiotics)	4 (1.5%)	7 (4.8%)	c2 = 4.012	FEp = 0.057
MI	0 (0.0%)	1 (0.7%)	c2 = 1.847	FEp = 0.352
DVT/PE	0 (0.0%)	0 (0.0%)	–	–
COVID	1 (0.4%)	2 (1.4%)	c2 = 1.314	FEp = 0.284
Re-operation	2 (0.7%)	0 (0.0%)	c2 = 1.091	FEp = 0.543
Death	2 (0.7%)	4 (2.7%)	c2 = 2.647	FEp = 0.190
Other	4 (1.5%)	9 (6.2%)	c2 = 6.823*	FEp = 0.015*
Re-attendance following initial assessment	21 (7.8%)	10 (6.8%)	c2 = 0.125	0.723
Operation	10 (47.6%)	3 (30.0%)	c2 = 0.864	FEp = 0.452
IR drain	2 (9.5%)	1 (10.0%)	c2 = 0.002	FEp = 1.000
Gastrografin	12 (57.1%)	8 (80.0%)	c2 = 1.546	FEp = 0.262
Mortality	28 (10.4%)	49 (33.6%)	c2 = 33.570*	>0.001*
30-day mortality	9 (3.3%)	22 (15.1%)	c2 = 18.816*	>0.001*
1-year mortality	10 (3.7%)	19 (13.0%)	c2 = 12.583*	>0.001*

Surgical group versus conservative only

To assess the outcomes, we must differentiate between the management approaches to be able to assess the outcomes of each approach, which is illustrated in Tables 5, 6.

**Table 5:** Comparison between operative and non-operative group *Statistically significant at p ≤ 0.05. SD: standard deviation, U: Mann–Whitney test, Min.: minimum, Max.: maximum, c2: chi-square test, FE: Fisher exact, p: probability value for comparison between the studied categories, BMI: body mass index, COPD: chronic obstructive pulmonary disease, ASA: American Society of Anaesthesiologists.

Parameters	Operation Not Required (Percentage) (n = 289)	Operation Required (Percentage) (n = 126)	Test of sig.	p-value
Length of hospital stay (days)				
Mean ± SD	6.93 ± 6.63	17.42 ± 16.94	U = 6207.000*	<0.001*
Median (Min–Max)	5.0 (.0–45.0)	12.0 (1.0–106.0)		
Sex				
Male	126 (43.6)	47 (37.3)	1.431	0.232
Female	163 (56.4)	79 (62.7)		
Comorbidity				
BMI > 40	10 (3.5)	4 (3.2)	1.022	FEp = 1.000
Diabetes	38 (13.1)	24 (19.0)	2.403	0.121
COPD	21 (7.3)	17 (13.5)	4.088*	0.043*
ASA				
I	40 (13.8)	6 (4.8)	16.328*	0.003*
II	106 (36.7)	40 (31.7)		
III	110 (38.1)	58 (46.0)		
IV	33 (11.4)	19 (15.1)		
V	0 (0.0)	3 (2.4)		
Frailty score				
Mean ± SD	4.51 ± 1.68	4.38 ± 1.69	U = 17420.500	0.477
Median (Min–Max)	4.0 (1.0–9.0)	4.0 (1.0–9.0)		
Therapeutic Gastrografin				
No	82 (28.4)	77 (61.1)	39.789*	<0.001*
Yes	207 (71.6)	49 (38.9)		

**Table 6:** Recurrence and mortality between operative vs non-operative groups Test of sig.: test of significance, c2: chi-square test, FE: Fisher exact, IR: interventional radiology, p: probability value for comparison between the studied categories. *Statistically significant at p ≤ 0.05.

Parameters	Operation Not Required (Percentage) (n = 289)	Operation Required (Percentage) (n = 126)	Test of sig.	p-value
Recurrence following initial management	29 (10.0)	2 (1.6)	9.059*	0.003*
Operation	13 (44.8)	0 (0.0)	1.544	FEp = 0.497
IR drain	3 (10.3)	0 (0.0)	0.229	FEp = 1.000
Gastrografin	18 (62.1)	2 (100.0)	1.176	FEp = 0.527
Mortality	58 (20.1)	19 (15.1)	1.446	0.229
30-day mortality	25 (8.7)	6 (4.8)	1.920	0.166
1-year mortality	20 (6.9)	9 (7.1)	0.007	0.935

The percentage of COPD patients was statistically and significantly higher in the operative group, which can be explained by the fact that COPD patients are likely to develop desaturation and respiratory distress with abdominal distention and intestinal obstruction. This is a very interesting result, showing that they required surgery more often than non-COPD patients. 

The American Society of Anesthesiologists (ASA) classification indicates a high level of comorbidity and complexity among the patients, with 168 (40.5%) classified as ASA III (severe systemic disease) and 52 (12.5%) as ASA IV (severe systemic disease that is a constant threat to life). These classifications reinforce the need for careful perioperative management and highlight the potential risks associated with surgical interventions in this population.

The data regarding the use of therapeutic Gastrografin in relation to the necessity for surgical intervention provide compelling insights into its effectiveness in managing ASBO. Among patients who did not require surgery, 82 (28.4%) were treated without Gastrografin, while a significant 207 (71.6%) were treated with Gastrografin. These findings highlight the therapeutic benefits of Gastrografin, suggesting that it not only aids in the diagnostic process but also enhances conservative management strategies for SBO. A total of 77 (61%) patients of the operative group were not offered Gastrografin because a decision had been made for surgical intervention due to clinical indications such as peritonitis, haemodynamic instability, refusal of NGT insertion during conservative treatment, or clinical suspicion of bowel ischaemia.

Complications were noted in the surgical group, with wound infections requiring antibiotics in 8 (6.3%) of patients and a similar rate needing drainage. Other complications included postoperative ileus in 20 (15.9%) and hospital-acquired pneumonia (HAP), with 11 (8.7%) requiring intravenous antibiotics. The low rates of serious complications and re-operation in 2 (0.5%) suggest that surgical interventions, while not without risk, were generally performed safely. None of the conservative groups developed HAP or aspiration pneumonia, which supports the clinical importance of keeping an NGT in place if conservative management is considered, as it helps avoid aspiration even if patients do not respond to conservative treatment. Patients who were managed non-operatively had a mean hospital stay of 6.93 days (± 6.63), while those who underwent surgery experienced a markedly longer mean stay of 17.42 days (± 16.94). The statistical evaluation yielded a U value of 6207.000, with a p-value of less than 0.001, indicating a highly significant difference between the two groups.

The median lengths of stay further emphasise this disparity, with non-operated patients having a median stay of 5.0 days compared to a median of 12.0 days for those who required surgical intervention. This suggests that surgical patients are not only hospitalised for longer periods but also face a greater variability in their recovery times. Our data showed that only 6 (4.7%) patients out of 126 (30.3%) in the surgical group had delayed surgical intervention (later than 72 hours from admission), which supports linking postoperative recovery time to the length of hospital stay, as most patients who had surgery underwent the procedure within the first 72 hours, meaning there was no preoperative delay.

Table 7 discusses the patients grouped under the various criteria who had to undergo surgery and the odds ratios of these criteria that help predict the need for surgical intervention.

**Table 7:** Relation between operation required and different parameters showing odds ratios for predictions of surgical interventions OR: odds ratio, CI: confidence interval, LL: lower limit, UL: upper limit. *Statistically significant at p ≤ 0.05.

Parameter	Operation Required (Q30a)		OR (LL–UL 95% CI)	p-value
	No (N = 289), n (%)	Yes (N = 126), n (%)		
Sex				
Male	126 (43.6)	47 (37.3)	-	1
Female	163 (56.4)	79 (62.7)	1.299 (0.846–1.996)	0.232
Comorbidity				
BMI >40	10 (3.5)	4 (3.2)	0.915 (0.281–2.974)	0.882
Diabetes	38 (13.1)	24 (19.0)	1.554 (0.887–2.7722)	0.123
COPD	21 (7.3)	17 (13.5)	1.990 (1.011–3.917)	0.046*
Current smoker	13 (4.5)	13 (10.3)	0.409 (0.184–0.911)	0.029*
Asthma	22 (7.6)	12 (9.5)	1.278 (0.611–2.669)	0.515
MI/stroke	37 (12.8)	16 (12.7)	0.991 (0.529–1.856)	0.977
Heart failure	26 (9.0)	9 (7.1)	0.778 (0.354–1.712)	0.533
Malignancy	93 (32.2)	22 (17.5)	0.446 (0.265–0.751)	0.002*
CKD	16 (5.5)	7 (5.6)	0.996 (0.399–2.485)	0.0994
Immunosuppression	20 (6.9)	8 (6.3)	0.912 (0.391–2.129)	0.831
Dementia	5 (1.7)	4 (3.2)	1.862 (0.492–7.054)	0.36
ASA				
I	40 (13.8)	6 (4.8)	-	1
II	106 (36.7)	40 (31.7)	2.516 (0.991–6.389)	0.052
III	110 (38.1)	58 (46.0)	3.515 (1.408–8.778)	0.007*
IV	33 (11.4)	19 (15.1)	3.838 (1.374–10.720)	0.010*
V	0 (0.0)	3 (2.4)	NA	-
Frailty score ≥ 5				
Mean ± SD	4.51 ± 1.68	4.38 ± 1.69	0.956 (0.843–1.083)	0.477
Median (Min–Max)	4.0 (1.0–9.0)	4.0 (1.0–9.0)		

Patients with COPD had roughly double the odds of requiring surgery compared with non-COPD patients. This relationship is statistically significant (p = 0.046). COPD is a relevant clinical risk factor for progression to operative management.

Patients with high ASA status (III-IV) had higher odds of requiring an operation compared with those with ASA I-II, and this was associated with a statistically significant p-value, which indicates that high ASA grade is therefore an important predictor of needing surgical intervention.

Although diabetes, asthma, and dementia showed high odds ratios of greater than 1, there was no significant p-value to confirm the prediction of the need for surgical intervention.

Table 8 compares operated cases who were given Gastrografin versus those who were not, and the length of stay is actually greater by two to three days for those who had a trial of Gastrografin preoperatively. This signifies that identifying surgical candidates is crucial, and anticipating failure of treatment based on radiological or clinical signs should be discussed. 

**Table 8:** Relation between therapeutic Gastrografin and different parameters for operated cases (n =126) SD: standard deviation, U: Mann–Whitney test, c2: chi-square test, FE: Fisher exact, Test of sig.: test of significance, HAP: hospital-acquired pneumonia, IV: intravenous. *Statistically significant at p ≤ 0.05.

Operated Cases (n = 126)	Therapeutic Gastrografin Not Given (n = 77)	Therapeutic Gastrografin Given (n = 49)	Test of sig.	p-value
Length of hospital stay (days)				
Mean ± SD	16.61 ± 17.99	18.69 ± 15.24	U = 1414.00*	0.018*
Complications				
Collection	3 (3.9%)	7 (14.3%)	c2 = 4.424*	FEp = 0.046*
HAP (requiring IV antibiotics)	10 (13.0%)	1 (2.0%)	c2 = 4.503*	FEp = 0.049*
Mortality	9 (11.7%)	10 (20.4%)	c2 = 1.778	0.182
30-day mortality	0 (0.0%)	6 (12.2%)	c2 = 9.900*	FEp = 0.003*
1-year mortality	7 (9.1%)	2 (4.1%)	c2 = 1.133	FEp = 0.480

Also, statistically, the overall mortality does not show any difference, but the 30-day mortality is higher in patients who had surgery after a failed Gastrografin trial, which may indicate earlier mortality. On further evaluation of the complications of surgery, there was no noticeable difference except for postoperative collection being more common in the Gastrografin group. This shows that the hospital stay was mainly a postoperative stay due to complications, a delayed decision to operate, or a delay caused by failure of the Gastrografin trial. This signifies why predictive factors of failure of conservative treatment are essential research topics to obtain more solid or objective data on which we can rely when making decisions to start conservative management.

We only had six patients who had a delayed operation (after 72 hours from admission) out of 126 patients operated on for ASBO. On the other hand, and as expected, patients who had Gastrografin preoperatively developed less HAP, although there is considerable questioning in the literature that Gastrografin increases the risk of aspiration.

Re-attendance after initial management was documented in 31 (7.5%) cases, with 13 (41.9%) of them requiring surgical intervention upon return. Notably, 20 (64.5%) of these patients were managed with Gastrografin, indicating its potential role in conservative management and follow-up treatment strategies. A re-attendance p-value of 0.003 strongly indicates a significant association between the initial management approach and the likelihood of experiencing recurrent obstruction. Only two out of 126 operated patients presented again with SBO, and both of them were treated successfully with Gastrografin. The conservative group showed recurrent bowel obstruction in 29 (10%), with 13 (44%) of them requiring surgery in the second admission.

On the other hand, there was no statistical difference in the overall mortality or the 30-day mortality between the postoperative group and the conservatively managed group. Mortality rates provide further insight into the patient outcomes. An overall up to three-year mortality rate in 77 patients (18.6%) is concerning, particularly with a 30-day mortality rate in 31 patients (7.5%). The one-year mortality rate in 29 patients (7.0%) also indicates that a significant proportion of patients experience long-term risks following treatment. This may reflect the underlying health conditions present in many of the patients, as highlighted in previous tables. The data regarding mortality rates reveal a stark contrast between the two age groups in the study. Among 28 (10.4%) patients younger than 75 years, the mortality rate was 10.4% (28 out of 269 patients), while in the 49 (33.6%) patients aged 75 and older, the mortality rate increased to 33.6% (49 out of 146 patients). The chi-square statistic of 33.570 with a p-value of less than 0.001 indicates a highly significant association between age and mortality. In conclusion, the significant difference in mortality rates between the two age groups highlights the need for targeted interventions and careful consideration of age-related factors in the management of ASBO, ultimately aiming to improve the survival outcomes for older patients.

## Discussion

The ASA classification indicates a high level of comorbidity and complexity among the patients, classified as ASA III (severe systemic disease) and ASA IV (severe systemic disease that is a constant threat to life) [7]. These classifications reinforce the need for careful perioperative management and highlight the potential risks associated with surgical interventions in this population.

A study in the *Journal of the American College of Surgeons* reported more than twofold mortality for elderly patients vs the young group undergoing emergency general surgery procedures [8]. Furthermore, the one-year mortality rate in 29 (7.0%) is consistent with reported data that suggest a concerning trend in mortality linked to ASBO [10].

The importance of postoperative care in managing SBO has also gained recognition. Enhanced recovery protocols and multidisciplinary approaches are being implemented to optimise recovery and minimise recurrence risks [11]. Our findings on the recurrence rates indicate that patients managed with a trial of non-operative management, 29 (10%), had a recurrence rate compared to only 2 (1.6%) for surgically managed patients. This discrepancy highlights the challenges associated with conservative management strategies, which have been shown to yield higher recurrence rates than surgical interventions [10]. Understanding these dynamics is essential for developing effective management strategies for patients with ASBO. Behman et al. (2019) reported recurrence rates of 20% for non-operative and 13% for operative patients.

Our findings illustrate a diverse patient population characterised by significant age and gender variation, alongside a high burden of comorbidities and frailty. Previous studies support the notion that patients with diabetes and other comorbidities experience more complications; specifically, diabetes has been associated with a higher incidence of postoperative complications, such as infections and longer recovery times [12]. This complexity necessitates a multidisciplinary approach to optimise patient outcomes and ensure comprehensive care.

In terms of surgical intervention, our study found that 126 (30.4%) of the 415 patients required surgery during their admission, either initially or after failed conservative management. This aligns with existing literature, which indicates that between 20% and 30% of patients with ASBO require operative treatment [13].

Considering the short-term mortality, our study’s 30-day mortality rate in 31 (7.5%) aligns with existing literature, which reports rates of 5% to 10% for patients with ASBO [14]. The significant difference in hospital stay lengths between operated and non-operated patients emphasises the complexities involved in managing ASBO. Our findings reveal a median length of stay of five days for non-operative patients compared to 12 days for those who underwent surgery, which illustrates the need for optimised treatment pathways that aim to improve patient outcomes while minimising hospital resource utilisation. Notably, each admission for SBO carries considerable costs and risks, with an average length of stay of around eight days and short-term mortality rates reported between 5% and 10% [15].

Conservative management strategies, including bowel rest, nasogastric tube (NGT) decompression, and intravenous fluid therapy, have gained emphasis, particularly for partial obstructions. Research indicates that approximately 75% of patients with SBO have intra-abdominal adhesions as the underlying cause, and up to 70% of these cases can be effectively managed conservatively in the absence of strangulation or bowel ischaemia [16]. This approach may help decrease the reliance on urgent surgical interventions and mitigate associated risks.

The mean frailty score of 4.47 in our study indicates that a significant portion of patients exhibit signs of frailty, which could negatively impact their recovery and overall outcomes following treatment. With a median frailty score of 4.0, ranging from 1.0 to 9.0, there is notable variability in patient health status, underscoring the necessity for tailored management strategies to address individual needs. Such frailty is often associated with poorer surgical outcomes and increased complications, emphasising the importance of preoperative assessments in this demographic group [17]. The mortality rate is notably higher in cases of complicated SBO, such as those involving bowel ischaemia, perforation, or sepsis. In these cases, the mortality rate can rise to 10% or higher. Mortality rates in these cases range from 10% to 40%, depending on the extent of bowel compromise and the speed of surgical intervention [18]. The application of water-soluble contrast agents in the management of SBO has been studied, showing that while it does not significantly reduce the necessity for surgical intervention (odds ratio 0.81, p = 0.300), it can shorten hospital stays for those who do not undergo surgery [19].

Our analysis of the ASA classification revealed a significant association between the severity of systemic disease and the need for surgical intervention. The chi-square statistic of 16.328, with a p value of 0.003, indicates that higher ASA scores correlate with increased surgical necessity. For instance, 168 (46.0%) of ASA III patients required surgery compared to only 46 (4.8%) of ASA I patients, highlighting the critical need for assessing patient health status preoperatively.

In elderly patients, decision-making regarding surgical interventions is often controversial due to the associated comorbidities, mortality risks, and prolonged hospital stays. Our data confirm higher mortality and complication rates in the elderly group compared to those under 75 years, but interestingly, age did not significantly influence the type of surgery performed. The p-value of 0.929 indicates a non-relevant association between age and surgical intervention necessity. This suggests that while older patients may experience more complications, their surgical management decisions remain unaffected by age alone.

Limitations of the study and further research recommendations

Our study represents a retrospective database analysis, in which unmeasured differences, such as the delay in timing of Gastrografin intervention carrying a negative effect on prognosis, the type of initial surgery and the number of previous surgeries affecting the outcomes, and having no clarity on the number of episodes of ASBO (primary and recurrent), and being single-centre based constrain implication and generalising to other geographical or institutional contexts. Would this affect the management outcomes?

All unmeasured differences could have given us a deeper idea about the predictive risks and expected outcomes of these risks to our patients. It would be beneficial to conduct a similar study on a multi-centric scale and in a prospective manner to achieve more robust and generalisable results.

## Conclusions

While surgical management appears to reduce the recurrence rates, it is associated with increased hospital stays and similar mortality outcomes when compared to conservative approaches. The data indicate a higher burden of postoperative care for those undergoing surgery. The outcomes confirm that conservative management strategies could be effective, particularly in patients with lower recurrence risk or those with significant comorbidities that could complicate surgical outcomes. Although choosing a trial of conservative management and delaying surgical management if conservative management fails can increase early mortality postoperatively, the data show that our management protocol is safe, and the outcomes align with the literature. On the other hand, we highlight that early identification of patients indicated for surgery without a trial of conservative management can decrease mortality and morbidity. Patients who are elderly or with multiple comorbidities are expected to be indicated for surgery either initially or after failure of conservative management. Elderly patients present higher mortality rates and the occurrence of major complications.

## References

[1] Menzies D, Parker M, Hoare R, Knight A (2001). Small bowel obstruction due to postoperative adhesions: treatment patterns and associated costs in 110 hospital admissions. Ann R Coll Surg Engl.

[2] Ten Broek RP, Krielen P, Di Saverio S (2018). Bologna guidelines for diagnosis and management of adhesive small bowel obstruction (ASBO): 2017 update of the evidence-based guidelines from the world society of emergency surgery ASBO working group. World J Emerg Surg.

[3] Di Saverio S, Coccolini F, Galati M (2013). Bologna guidelines for diagnosis and management of adhesive small bowel obstruction (ASBO): 2013 update of the evidence-based guidelines from the world society of emergency surgery ASBO working group. World J Emerg Surg.

[4] NELA Project Team (2019). Fifth patient report of the National Emergency Laparotomy Audit. Fifth Patient Report of the National Emergency Laparotomy Audit.

[5] Ceresoli M, Coccolini F, Catena F, Montori G, Di Saverio S, Sartelli M, Ansaloni L (2016). Water-soluble contrast agent in adhesive small bowel obstruction: a systematic review and meta-analysis of diagnostic and therapeutic value. Am J Surg.

[6] Branco BC, Barmparas G, Schnüriger B, Inaba K, Chan LS, Demetriades D (2010). Systematic review and meta-analysis of the diagnostic and therapeutic role of water-soluble contrast agent in adhesive small bowel obstruction. Br J Surg.

[7] Doyle DJ, Hendrix JM, Garmon EH (2017). American Society of Anesthesiologists Classification (ASA Class). Statpearls [Internet].

[8] Ingraham AM, Cohen ME, Raval MV, Ko CY, Nathens AB (2011). Variation in quality of care after emergency general surgery procedures in the elderly. J Am Coll Surg.

[9] Köstenbauer J, Truskett PG (2018). Current management of adhesive small bowel obstruction. ANZ J Surg.

[10] Behman R, Nathens AB, Mason S, Byrne JP, Hong NL, Pechlivanoglou P, Karanicolas P (2019). Association of surgical intervention for adhesive small-bowel obstruction with the risk of recurrence. JAMA Surg.

[11] Barbois S, Arvieux C (2023). Principles of emergency and trauma laparotomy. Textbook of Emergency General Surgery: Traumatic and Non-traumatic Surgical Emergencies.

[12] Karamanos E, Dulchavsky S, Beale E, Inaba K, Demetriades D (2016). Diabetes mellitus in patients presenting with adhesive small bowel obstruction: delaying surgical intervention results in worse outcomes. World J Surg.

[13] Maienza E, Godiris-Petit G, Noullet S, Menegaux F, Chereau N (2023). Management of adhesive small bowel obstruction: the results of a large retrospective study. Int J Colorectal Dis.

[14] Behman R, Nathens AB, Haas B, Look Hong N, Pechlivanoglou P, Karanicolas P (2019). Population-based study of the impact of small bowel obstruction due to adhesions on short- and medium-term mortality. Br J Surg.

[15] Olausson M, Aerenlund MP, Azzam M (2023). Management and short-term outcomes of patients with small bowel obstruction in Denmark: a multicentre prospective cohort study. Eur J Trauma Emerg Surg.

[16] Podda M, Khan M, Di Saverio S (2021). Adhesive small bowel obstruction and the six w’s: who, how, why, when, what, and where to diagnose and operate?. Scand J Surg.

[17] Kehlet H, Wilmore DW (2008). Evidence-based surgical care and the evolution of fast-track surgery. Ann Surg.

[18] Schick MA, Kashyap S, Collier SA, Meseeha M (2025). Small bowel obstruction. Statpearls [Internet].

[19] Goussous N, Eiken PW, Bannon MP, Zielinski MD (2013). Enhancement of a small bowel obstruction model using the gastrografin® challenge test. J Gastrointest Surg.

